# A Great Book

**Published:** 1875-08

**Authors:** 


### A GREAT BOOK.

A ponderous volume, containing about as many pages as the world has seen years since the Christian era, has been sent to our office by the publisher. It is a Business Directory of the United States ; that is to say, it purports to give, upon its ample leaves, the names of all business men of importance, in all towns and cities in the country having a population of three thousand or more.

Of course, the labor involved in such a work is enormous, but it is by no means impossible. No one man could do it, nor any score of men. Our business populations change so often and so rapidly that a work of this class can only be made to approach accuracy by employing a large force, and by bringing down the canvassing to the very last moment before going to press.

Errors will occur, however, in all works of this class—errors of omission as well as of commission. Perhaps they are fewer in this great book than would naturally be expected. We turn to places concerning which our knowledge is accurate, and we find evidences of careful canvassing. We conclude that every section represented has been actually visited by a representative of the publisher, and that local directories have not been depended upon to any great extent.

The publisher of this enormous volume is Mr. T. Elwood Zell, of Philadelphia, who supplies New York buyers of the book through Messrs. Jacocks & Tappin, 91 Duane street. Mr. Zell has had large experience in this class of work, having long been engaged in the publication of popular Encyclopedias. In such a school he has unquestionably acquired the wonderful patience which is so essential to success in the compilation of such a book.

To the banker, the manufacturer, the merchant, the wholesale dealer, the jobber, the commission merchant, the retailer, the insurance man, the broker, and the professional man, this work is a necessity, as it renders communication with fellow-workers in any of these lines, easy. The publisher of periodical literature, who desires to communicate with newsdealers will have to look further for the names of those in the trade. This omission is to us, as publishers, a serious one, and we hope to see it corrected in the edition for 1876. In general, however, the book pleases us, and we should find ourselves consulting it several times a day, if our neighbors, the Health Food Company, would borrow it less constantly. They find the names of the grocers of the nation in the volume, and make use of it to mail to them circulars descriptive of their Granulated Wheat Biscuit. To them the work proves invaluable, as it must to all who seek to make their business known by means of circulars.

